# MYCN in human development and diseases

**DOI:** 10.3389/fonc.2024.1417607

**Published:** 2024-05-31

**Authors:** Yosuke Nishio, Kohji Kato, Hisashi Oishi, Yoshiyuki Takahashi, Shinji Saitoh

**Affiliations:** ^1^ Department of Pediatrics and Neonatology, Nagoya City University Graduate School of Medical Sciences, Nagoya, Japan; ^2^ Department of Pediatrics, Nagoya University Graduate School of Medicine, Nagoya, Japan; ^3^ Department of Genetics, Research Institute of Environmental Medicine, Nagoya University, Nagoya, Japan; ^4^ Department of Comparative and Experimental Medicine, Nagoya City University Graduate School of Medical Sciences, Nagoya, Japan

**Keywords:** MYCN, megalencephaly, polydactyly, Feingold syndrome, human development

## Abstract

Somatic mutations in *MYCN* have been identified across various tumors, playing pivotal roles in tumorigenesis, tumor progression, and unfavorable prognoses. Despite its established notoriety as an oncogenic driver, there is a growing interest in exploring the involvement of MYCN in human development. While *MYCN* variants have traditionally been associated with Feingold syndrome type 1, recent discoveries highlight gain-of-function variants, specifically p.(Thr58Met) and p.(Pro60Leu), as the cause for megalencephaly-polydactyly syndrome. The elucidation of cellular and murine analytical data from both loss-of-function (Feingold syndrome model) and gain-of-function models (megalencephaly-polydactyly syndrome model) is significantly contributing to a comprehensive understanding of the physiological role of MYCN in human development and pathogenesis. This review discusses the MYCN’s functional implications for human development by reviewing the clinical characteristics of these distinct syndromes, Feingold syndrome, and megalencephaly-polydactyly syndrome, providing valuable insights into the understanding of pathophysiological backgrounds of other syndromes associated with the MYCN pathway and the overall comprehension of MYCN’s role in human development.

## Introduction

*MYCN* belongs to the MYC proto-oncogene family, alongside *MYC* and *MYCL* (1). As transcription factors, MYC proteins govern the diverse genes’ expression pivotal in fundamental cellular biological functions such as proliferation, differentiation, and apoptosis (2, 3). Initially identified as a proto-oncogene amplified in neuroblastoma, *MYCN* amplification occurs in 20%-30% of neuroblastoma cases, serving as a critical prognostic marker associated with poorer outcomes (4). Furthermore, *MYCN* amplification and single nucleotide variants have been identified in various other tumors, encompassing Wilms’ tumor, rhabdomyosarcomas, and lung cancers (5–8).

MYCN and other members of the MYC protein family are expressed in various developing human fetal tissues, including the brain, limbs, heart, kidney, and lungs as well as within tumors (9–12). Notably, consistent with these expression patterns, heterozygous loss-of-function (LoF) *MYCN* variants have harmful effects on fetal development, giving rise to a genetic syndrome marked by multiple congenital anomalies known as Feingold syndrome type 1 (FS1, OMIM #164280) (13).

In addition to the direct *MYCN* variants, variations in genes associated with the physiological upstream pathways of MYC family, Wnt/β-catenin (14) and sonic hedgehog (SHH) pathways (15–17), contribute to various human developmental diseases (18–24). Furthermore, genes associated with receptor tyrosine kinase (RTK) signaling pathway (25, 26) and F-box and WD repeat domain containing 7 (*FBXW7*) (27) that contribute to the modulation of stability and activity of MYCN, *cyclin D*, one of the downstream transcribed genes of MYCN, and MYC associated factor X (*MAX*), a component of the MYC/MAX complex are documented as the causes of human developmental diseases (28–33). Though these increasing evidence for the association of human developmental diseases with MYCN is being established, the direct functional roles of MYCN on human development remain to be understood comprehensively.

Recently, we established the megalencephaly-polydactyly syndrome (OMIM #620748) in three individuals harboring heterozygous gain-of-function (GoF) *MYCN* variants, namely p.(Thr58Met) and p.(Pro60Leu), that helped understanding the MYCN’s roles in human development. Intriguingly, this syndrome manifested mirror phenotypes of FS1, featuring megalencephaly and postaxial polydactyly, complicated with neuroblastoma detected in two out of the three patients (34, 35). Functional analysis unveiled excessive stability and reduced phosphorylation at the 58th threonine residue, a crucial locus for MYCN degradation. Insights from the GoF mouse model highlighted the overproliferative tendencies of neuronal progenitors and potentially various tissues, resulting in megalencephaly and the involvement of several organs (34, 35).

Our review mainly focuses on the disorders directly related to *MYCN* to unravel MYCN’s functional roles in human development through reviewing cellular and murine models representing both LoF and GoF scenarios, along with the concise clinical descriptions of other syndromes associated with the MYCN pathway for the overall comprehension of MYCN’s roles in human development.

## MYCN as oncogenic drivers in various tumors

Dysregulation of MYCN is observed in various tumors in both pediatric and adult settings, encompassing neuroblastoma, Wilms’ tumor, rhabdomyosarcoma, lung cancer, medulloblastoma, retinoblastoma and basal cell carcinoma (5–8, 36–41). The oncogenic variants include amplification and somatic single nucleotide mutations that stabilize or activate MYCN.

Firstly, *MYCN* amplification is well established in neuroblastoma accounting for 20%-30% of cases (4), but also observed in 25% of alveolar rhabdomyosarcoma cases (4, 41, 42), 5%-10% of medulloblastomas associated with poor prognosis (36, 40). Among those tumors, medulloblastoma is grouped into four distinct molecular subgroups in terms of gene expression patterns, one of which is SHH group (43), in which the amplifications of *MYCN* and *MYCL* are most frequently observed, defining its pathological significance in medulloblastoma (44). In terms of molecular background, the amplification drives tumorigenesis in neural crest cells by maintaining or re-establishing embryonic characteristics such as self-renewal, apoptotic resistance or metabolic flexibility (45). From a murine study with a transgenic mouse expressing MYCN targeted to the neural crest (TH-MYCN mice), the mouse develops neuroblastoma that begins with hyperplastic lesions in paravertebral ganglia within the first weeks after birth, escaping from the normal physiological process of cell death (46). This explains one of the etiologies of tumorigenesis due to amplification, though it should be kept in mind that not all gene amplification result in high level of gene expression (47, 48).

Secondly, somatic single nucleotide mutations, most frequently p.(Pro44Leu) (P44L), are identified in varieties of tumors such as neuroblastoma, medulloblastoma, and Wilms’ tumor (49–51). Among those, it is noteworthy that the P44L mutation is observed as frequently as 1.7% of high-risk neuroblastoma without MYCN amplification (49). Although the functional consequences of the P44L mutation remained unclear until recently, the significantly slower decay of MYCN was observed in OP9-DL1 cells transduced with lentiviral vectors expressing MYCN-P44L (52). Although the 44^th^ proline is located adjacent to the conserved phosphordegron site by FBXW7, the interaction with FBWX7 is not altered in MYCN-P44L, that keeps the molecular background of the active MYCN function under investigations (53). In addition to the P44L variant, the mutationally stabilized variant, p.(Thr58Ala) (T58A), is analyzed using neural stem cells (NSCs) transduced with the variant. Transplantation of N-myc^T58A^ embryonic cerebellar NSCs develops SHH-dependent medulloblastoma (54). Collectively, MYCN functions as a key oncogenic driver in various tumors.

## Spatiotemporal expression of MYCN during development

For understanding the essential roles of MYCN in human developmental diseases as well as oncogenesis, the spatiotemporal expression patterns during development should be emphasized. In terms of the temporal aspect, Mycn is highly expressed at embryonic day (E) 13.5, then gradually decreased to its lowest level by postnatal day (P) 15 in mice, suggesting its important roles during embryo- and organogenesis (34). Expanding the focus on the spatial aspect, RNA *in situ* hybridization and Northern blot analysis of murine embryos suggest the extensive expression profiles of Mycn. Although Myc transcripts are expressed in various tissues at lower levels, Mycn expression indicates the tissue- and cell-specific patterns (55), preferring epithelial tissues, highest in the central nervous system, cranial and spinal ganglia in the peripheral nervous system, and heart (55). In addition, the transcripts are found in the developing gut, kidney, and lung. Interestingly, Myc transcripts are restricted to the mesenchymal compartments, rather than the epithelium, indicating interconnected regulatory mechanisms governing embryogenesis (55). Overall, these spatiotemporal expression profiles during development suggest its pivotal role in normal developmental processes beyond the context of tumorigenesis and progression.

## MYCN and associated pathway

**Figure 1:**
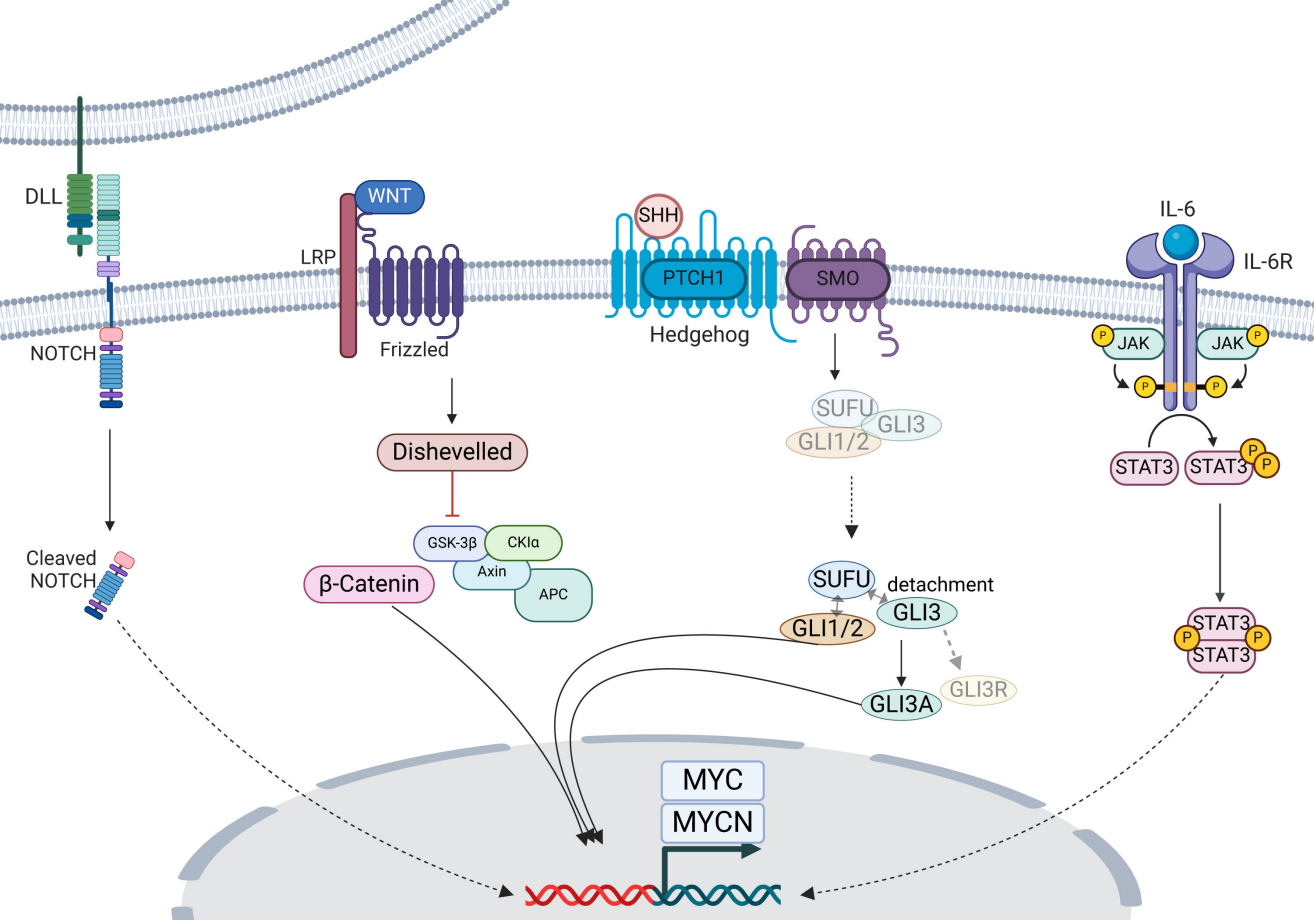
Overview of MYCN-upstream pathway. The Sonic Hedgehog (SHH) and Wnt/β-catenin pathway boosts MYCN transcription, whereas Notch and IL6/JAK/STAT3 pathway regulate MYC transcription. DLL, delta-like; LRP, LDL receptor-related protein. The figure was created with BioRender.com.

MYCN and other MYC family are regulated by various upstream pathways (**Figure 1**). Among those are Wnt/β-catenin and SHH pathways that directly target MYCN (14–17), whereas Notch and IL6-JAK-STAT3 pathways are established for the upstream pathways in the context of MYC but not yet of MYCN (56, 57). Wnt signaling pathway initiates with the binding of Wnt ligand to the frizzled receptor and LDL receptor-related protein (LRP) co-receptor. Though GSK-3, CK-1α, Axin, and APC complex with and degrade β-catenin in the absence of the ligand, the binding of the ligand initiates the disruption of the protein complex. Then, dissociated from the complex, accumulates in cytoplasm, subsequently translocating into the nucleus, where it couples with T cell factor/lymphoid enhancer factor, initiating the transcription of targets genes, including *MYC* and *MYCN* (58). Speaking of SHH pathway, the sonic hedgehog signaling molecule (SHH) serves as the principal inductive ligand, crucial for shaping the ventral neural tube, determining the anterior-posterior axis of limbs, and influencing the patterning of ventral somites (59, 60). Briefly, SHH initiates a cascade of events by binding to its receptor, patched 1 (PTCH1), relieving its inhibition on smoothened, frizzled class receptor (SMO) and leading to the release of sufu negative regulator of hedgehog signaling (SUFU) from GLI family zinc finger 1, 2, and 3 (GLI1, 2, and 3) (61, 62). This process mediates the intranuclear transition of GLI1 and 2 and prevents GLI3 cleaved into the repressor form, GLI3R, together promoting the GLI1, 2 and GLI3A (the active form) mediated-expression of SHH target genes, including *MYCN* (15, 16).

**Figure 2:**
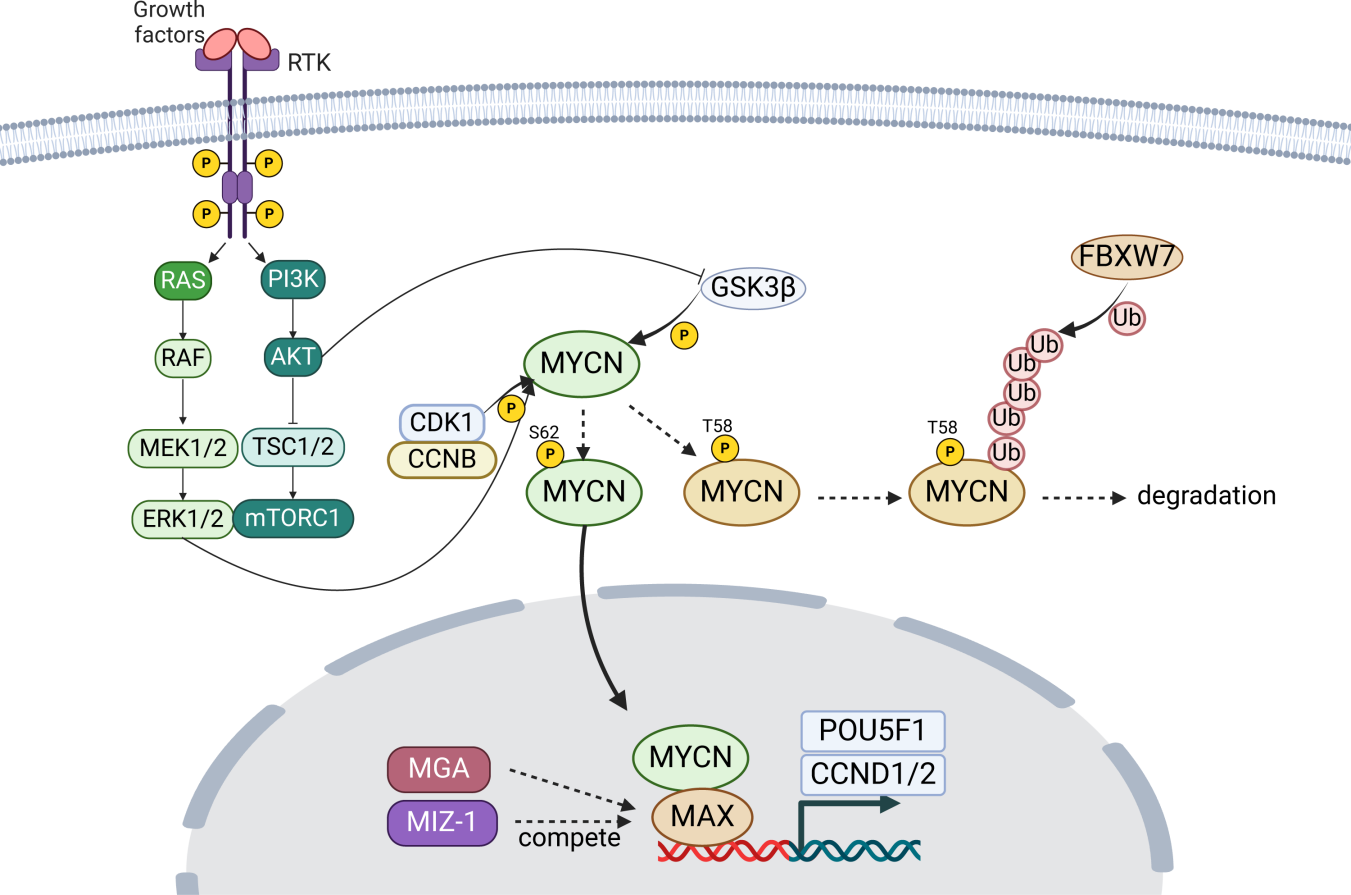
Overview of MYCN stabilization and activity. MYCN targets genes like *POU5F1*, essential for cell renewal, and promoting proliferation through genes *CCND1* and *CCND2*. Stability of MYCN is modulated by phosphorylation, affecting its degradation or stabilization. RTK, receptor tyrosine kinase; P, phosphate; Ub, ubiquitin. The figure was created with BioRender.com.

MYCN itself regulates cellular proliferation and differentiation by functioning as a transcription factor (**Figure 2**). Specifically, MYCN, coupled with MAX, controls these processes by activating or inhibiting the transcription of specific genes (63–66). This dimerization processes are competed by DNA-binding counterparts (e.g., MAX dimerization protein (MGA) and zinc finger- and BTB domain-containing protein 17 (ZBTB17, or MIZ-1)) (67, 68), which are essential for early development demonstrated by embryonic lethality of biallelic inactivation alleles though they have not been established as the cause of human diseases (69, 70). For transcriptional targets, *POU5F1* is a critical gene involved in cellular self-renewal and differentiation. Studies have shown that MYCN directly binds to the enhancer region of the *POU5F1* and enhances its transcription (27, 71). Consequently, POU5F1 engages with the promoter regions of *CCND1* and *CCND2*, critical transcription factors controlling the transition from the G1 phase to the S phase, thereby facilitating cellular growth and proliferation (72, 73). Collectively, MYCN plays a significant role in modulating cells’ gene expression profile through such mechanisms, thereby regulating cellular proliferation and differentiation.

In addition to the canonical targets, dysregulated MYCN expression also promotes binding to noncanonical (CANNTG) E-boxes clustered in enhancers, so called, “enhancer invasion” (74). When the expression of MYCN is physiological, it binds predominantly to canonical promoter E-boxes, but the excess MYCN spreads out, binding weaker E-box enhancer sites. The expression of genes with MYCN-invaded enhancers depends on the tissue-specific transcription factor, such as TWIST1 in neuroblastoma, defining highly tumor-specific ‘MYC target gene signatures’, expanding from the canonical targets’ origined signature (74).

In terms of modulating MYCN stability, the phosphorylation at Thr58 (T58) by glycogen synthase kinase three beta (GSK3β) that is regulated by PI3K/AKT pathway, following the phosphorylation at Ser62 (S62) by cyclin-dependent kinase 1 (CDK1) and ERK1/2, plays a crucial role (25, 26, 75, 76) (**Figure 2**). The phosphorylation at T58 destabilizes the MYCN protein, whereas that of S62 stabilizes it. Accordingly, dephosphorylation at T58 stabilizes the protein, preventing its degradation, following FBXW7-mediated ubiquitination (75, 76).

Collectively, the dynamic interaction between MYCN and associated pathways highlights the sophisticated regulatory networks governing cellular development, underscoring the complexity of developmental biology and the potential implications for understanding and treating developmental disorders.

## MYCN upstream pathways and human developmental diseases

Human disorders, including developmental diseases and tumors, associated with MYCN upstream pathways, SHH and WNT/β-catenin pathways, are well established so far. Although the contribution of MYCN, their downstream target, to the pathogenesis cannot be ignored to discuss the overall pathophysiology, it remains unclear because of their varieties of effects with multiple targets aside from MYCN. For imagining the potential MYCN’s roles in clinical consequences of those disorders through this review, we first introduce disorders associated with those two important upstream pathways (summarized in **Table 1**).

**Table 1:** MYCN upstream pathway and human developmental diseases.

	Phenotype	MIM number	Causative gene	Inheritance	Functional alteration	Transcriptional activity of MYCN
Sonic hedgehog	Holoprosencephaly 3	#142945	*SHH*	AD	LoF	?
	Single median maxillary central incisor	#147250		AD	?	?
	Microphthalmia with Coloboma 5	#611638		AD	?	?
	Basal cell nevus syndrome 1	#109400	*PTCH1*	AD	LoF	?
	Holoprosencephaly 7	#610828		AD	GoF?	?
	Basal cell nevus syndrome 2	#620343	*SUFU*	na	?	?
	Joubert syndrome 32	#617757		AR	LoF	?
	Polydactyly, postaxial, type A8	#618123	*GLI1*	AR	LoF	?
	Polydactyly, preaxial I	#174400		AR	LoF	?
	Culler-Jones syndrome	#615849	*GLI2*	AD	LoF	?
	Holoprosencephaly 9	#610829		AD	LoF	?
	Greig cephalopolysyndactyly syndrome	#175700	*GLI3*	AD	LoF	?
	Pallister-Hall syndrome	#146510		AD	?	?
	Polydactyly, postaxial, types A1 and B	#174200		AD	LoF	?
	Polydactyly, preaxial, type IV	#174700		AD	LoF	?
Wnt/β-catenin	Osteogenesis imperfecta, typeXV	#615220	*WNT1*	AR	LoF	?
	Mullerian aplasia and hyperandrogenism	#158330	*WNT4*	AD	LoF	?
	Robinow syndrome, autosomal dominant 1	#180700	*WNT5A*	AD	LoF	?
	Fuhrmann syndrome	#228930	*WNT7A*	AR	LoF	?
	Schopf-Schulz-Passarge syndrome	#224750	*WNT10A*	AR	LoF	?
	Ectodermal dysplasia 16 (odontoonychodermal dysplasia)	#257980		AR	LoF	?
	Split-hand/foot malformation 6	#225300	*WNT10B*	AR	LoF	?
	Omodysplasia 2	#164745	*FZD2*	AD	LoF	?
	Exudative vitreoretinopathy 1	#133780	*FZD4*	AD	LoF	?
	Microphthalmia/coloboma 11	#620731	*FZD5*	AD	LoF	?
	Robinow syndrome, autosomal recessive	#268310	*ROR2*	AD	LoF	?
	Robinow syndrome, autosomal dominant 2	#616331	*DVL1*	AD	LoF	?
	Robinow syndrome, autosomal dominant 3	#616894	*DVL3*	AD	LoF	?

AD, autosomal dominant; AR, autosomal recessive; LoF, loss of function; GoF, gain of function.

The inconclusive information is indicated with question marks.

### Sonic hedgehog pathway

SHH is associated with three distinct disorders: holoprosencephaly 3 (HPE3, OMIM #142945), single median maxillary central incisor (SMMCI, OMIM #147250) and microphthalmia with coloboma 5 (OMIM #611638). HPE3 is a severer spectrum of the failure for the developing forebrain to correctly segregate into distinct right and left hemispheres (77). In contrast, SMMCI manifests with the milder form, presenting only one deciduous and one permanent maxillary central incisor, often accompanied by short stature (78, 79). Furthermore, microphthalmia with coloboma 5 involves bilateral microphthalmia with inferonasal chorioretinal coloboma (78–80). Though the genotype-phenotype relationship has not been established, mutations in a long-range enhancer located upstream of *SHH* disrupt limb patterning, leading to the development of preaxial polydactyly (81).

Heterozygous mutations in *PTCH1* (patched 1) have been identified in patients with Gorlin syndrome, also known as basal cell nevus syndrome 1 (BCNS1, OMIM #109400), and holoprosencephaly 7 (HPE7, OMIM #610828). BCNS1 is characterized by basal cell carcinomas, epidermal cysts, calcified dural folds, jaw keratocysts, palmar and plantar pits, ovarian fibromas, medulloblastomas, lymphomesenteric cysts, fetal rhabdomyoma, and various signs of maldevelopment, including macrocephaly (20, 21). HPE7, characterized by holoprosencephaly with incomplete penetrance, seems to be caused by the variants, acquiring enhanced repressive activity on the SHH pathway (19, 82).

Homozygous LoF variants within *SUFU* (SUFU negative regulator of hedgehog signaling) are identified in patients diagnosed with Joubert syndrome 32 (OMIM #617757), characterized by global developmental delay, intellectual disability, dysmorphic facial features, and minor anomalies such as postaxial polydactyly (22). In addition, germline variants in *SUFU* are found among patients affected with basal cell nevus syndrome 2 (BCNS2, OMIM #620343) (23, 83).

GLIs (GLI family zing fingers) are critical transcription factors, categorized into GLI1, 2, and 3. The *GLI1* variants have been identified as the causes of polydactyly, preaxial I (OMIM #174400) and polydactyly, postaxial, type A8 (OMIM #618123) with an autosomal recessive manner (84, 85). Furthermore, mutations or dysregulation of *GLI2* are linked to multiple human developmental diseases, such as Culler-Jones syndrome (OMIM #615849), characterized by hypopituitarism, abnormalities of the external genitalia, and postaxial polydactyly (86) and holoprosencephaly 9 (HPE9, OMIM #610829) (87). In addition, the variants in *GLI3* have been identified in patients of Greig cephalopolysyndactyly syndrome (GCPS, OMIM #175700) and Pallister-Hall syndrome (PHS, OMIM #146510). The former is characterized by frontal bossing, scaphocephaly, and hypertelorism, alongside pre- and postaxial polydactyly with syndactyly (88), whereas, the latter manifests with hypothalamic hamartoma, pituitary dysfunction, central polydactyly, and visceral malformations (89). *GLI3* mutations also cause isolated pre- and postaxial polydactyly (OMIM #174200) (90, 91).

### Wnt/β-catenin pathway

WNTs (Wingless-type MMTB integration site family), a family of secreted glycoproteins, are associated with varieties of developmental diseases (24): WNT1 for osteogenesis imperfecta, type XV characterized by bone fragility and low bone mass with developmental delay and brain malformation (OMIM#615220), WNT4 for mullerian aplasia and hyperandrogenism by aplasia of mullerian duct derivatives (OMIM#158330), WNT5A for Robinow syndrome, autosomal dominant 1 by dysmorphic features, mesomelic limb shortening, hypoplastic external genitalia in males, and renal and vertebral anomalies (OMIM#180700), WNT7A for Fuhrmann syndrome by skeletal anomalies including polydactyly (OMIM#601570), WNT10A for Schopf-Schulz-Passarge syndrome (OMIM#224750), ectodermal dysplasia 16 (odontoonychodermal dysplasia) (OMIM#257980), and WNT10B for split-hand/foot malformation 6 (OMIM#225300).

The receptors for the WNT ligand (FZD, frizzled class receptor) are also associated with various developmental diseases: FZD2 for omodysplasia 2 (OMIM#164745), FZD4 for exudative vitreoretinopathy 1 (OMIM#133780), FZD5 for microphthalmia/coloboma 11 (OMIM#620731), and ROR2 for Robinow syndrome, autosomal recessive (OMIM#268310). Furthermore, DVL1 and 3, encoding dishevelled, an intracellular scaffolding protein that act downstream of transmembrane WNT receptors, have been identified in the patients with Robinow syndrome 2 and 3 (OMIM#616331 and #616894).

## MYCN and downstream target in human developmental diseases

### MYCN

*MYCN* variants have been linked to two distinct human developmental diseases: FS1 and megalencephaly-polydactyly syndrome (summarized in **Table 2**).

**Table 2:** MYCN, CCND2, and associated genes and human developmental diseases.

	Phenotype	MIM number	Causative gene	Inheritance	Functional alteration	Transcriptional activity of MYCN
MYCN	Feingold syndrome 1	#164280	*MYCN*	AD	LoF	↓
	Megalencephaly-polydactyly syndrome	#620748		AD	GoF	↑
CCND	Megalencephaly-polymicrogyria-polydactyly-hydrocephalus syndrome 3	#615938	*CCND2*	AD	GoF	na
Others	Polydactyly-macrocephaly syndrome	#620712	*MAX*	AD	GoF	↑?
	Developmental delay, hypotonia, and impaired language	#620012	*FBXW7*	AD	LoF	↓?

AD, autosomal dominant; AR, autosomal recessive; LoF, loss of function; GoF, gain of function.

The upward and downward arrows indicates up- and down-regulation, respectively. The inconclusive information is indicated with question marks.

FS1 is characterized by a range of congenital anomalies, including digital anomalies (such as absent or hypoplastic phalanges), microcephaly, dysmorphic facial features, gastrointestinal atresia, and intellectual disability (13). Occasional findings may also include renal and cardiac abnormalities, along with hearing impairment (13). LoF variants in *MYCN* have been identified as the cause for this syndrome (92, 93).

In contrast to FS1 phenotypes, a *de novo MYCN* missense variant, p.(Thr58Met), is identified in a 15-year-old patient with megalencephaly, postaxial polydactyly, and neuroblastoma (34). The variant is located at the phosphorylation site in the MYC box 1, highly conserved, and absent in any population database (eg, gnomAD), although present in the database of somatic mutations in human cancers (eg, COSMIC). Functional analysis demonstrated the GoF properties of the identified variant (discussed later). Additionally, two additional cases involving a fetus and an 8-month-old patient, both of whom presented with remarkably similar phenotypes, including megalencephaly and postaxial polydactyly, with neuroblastoma observed in the latter, is documented to have heterozygous GoF *MYCN* variants, namely p.(Thr58Met) and p.(Pro60Leu) (35). The novel variant, p.(Pro60Leu), is located just near the phosphorylation site (the 58^th^ threonine) in the MYC box 1, highly conserved with a damaging *in silico* prediction, and absent in any population database (eg, gnomAD), despite present in the database of somatic mutations in human cancers (eg, COSMIC). Notably, these patients exhibited a reduced amount of white matter and ventriculomegaly, differentiating this condition from other megalencephalic syndromes, especially those linked to disturbances in the mTOR pathway (31–33). Interestingly, the phenotypic characteristics of this novel syndrome mirrored those of FS1.

### CCND2

*CCND2* GoF variants have been associated with megalencephaly-polymicrogyria-polydactyly-hydrocephalus syndrome 3 (MPPH3, OMIM#615938), a developmental brain disorder characterized by megalencephaly, bilateral perisylvian polymicrogyria, and postaxial polydactyly (28).

## Other MYCN-related genes

There are several MYCN-related genes established as the causative genes for human developmental diseases. GoF mutations in *MAX* are implicated in polydactyly-macrocephaly syndrome (PDMCS, OMIM#620712), characterized by postaxial polydactyly, progressive macrocephaly, ocular anomalies, and neurodevelopmental issues (29). The *FBXW7* is identified as the causative gene for developmental delay, hypotonia, and impaired language (OMIM #606278), characterized by global developmental delay, delayed speech, and distinctive facial features (30).

## Cellular and murine model

### MYCN loss-of-function model

A germline knockout mouse is embryonic lethal, dying around E11.5, accompanied by notable organ abnormalities in the nervous system, mesonephros, lung, and gut, consistent with its expression pattern (55). Expanding the focus on neural crest-specific Mycn conditional knockout mice, the targeted removal of Mycn impaired the proliferation of granule neuron progenitors (GNPs), disrupted foliation, and resulted in a diminished cerebellar mass (94–96). As suggested in conventional knockout embryos, the increased Myc mRNA levels in Mycn-null GNPs were observed, and the simultaneous deletion of both Myc and Mycn worsened the impairment in cerebellar development (94–96). Furthermore, Mycn deficiency induced the premature expression of two cyclin-dependent kinase inhibitors, Kip1 and Ink4c, within the cerebellar primordium (94). Disrupting the Kip1 and Ink4c genes in Mycn-null cerebella partially restores GNPs proliferation and cerebellar foliation, providing conclusive genetic evidence supporting the assertion that Mycn expression, along with the concurrent down-regulation of Ink4c and Kip1, significantly contributes to the proper developmental trajectory of the cerebellum. In addition to its extensive analyses of the central nervous system, skeletal mesenchymal stem cell-specific Mycn conditional knockout mice were generated as models for Feingold syndrome (97). These mice, recapitulating the human phenotypes of limb abnormalities, revealed another dimension of significance during embryo- and organogenesis. Although the expression of miR-17-92, which is coded by *MIR17HG*, the causative gene of Feingold syndrome type 2, is controlled with transcriptional regulation by MYCN, the study demonstrated the distinct molecular mechanisms between Feingold syndrome type 1 and 2 (97). Interestingly, Mir17-92 deficiency leads to the upregulation of TGF-β signaling, while Mycn deficiency induces the downregulation of PI3K signaling in limb mesenchymal cells. In addition, skeletal anomalies arising from Mir17-92 deficiency can be effectively ameliorated through genetic or pharmacological inhibition of TGF-β signaling, indicating a crucial role for elevated TGF-β signaling in the skeletal abnormalities of Feingold syndrome type 2, but the skeletal phenotype associated with Mycn-deficiency experiences only partial mitigation through Pten heterozygosity and fails to respond to TGF-β inhibition (97). These findings strongly emphasize that, despite phenotypic similarities, distinct and complicated molecular mechanisms govern the maldevelopment of Feingold syndrome types 1 and 2 (97).

### MYCN gain-of-function model

**Figure 3:**
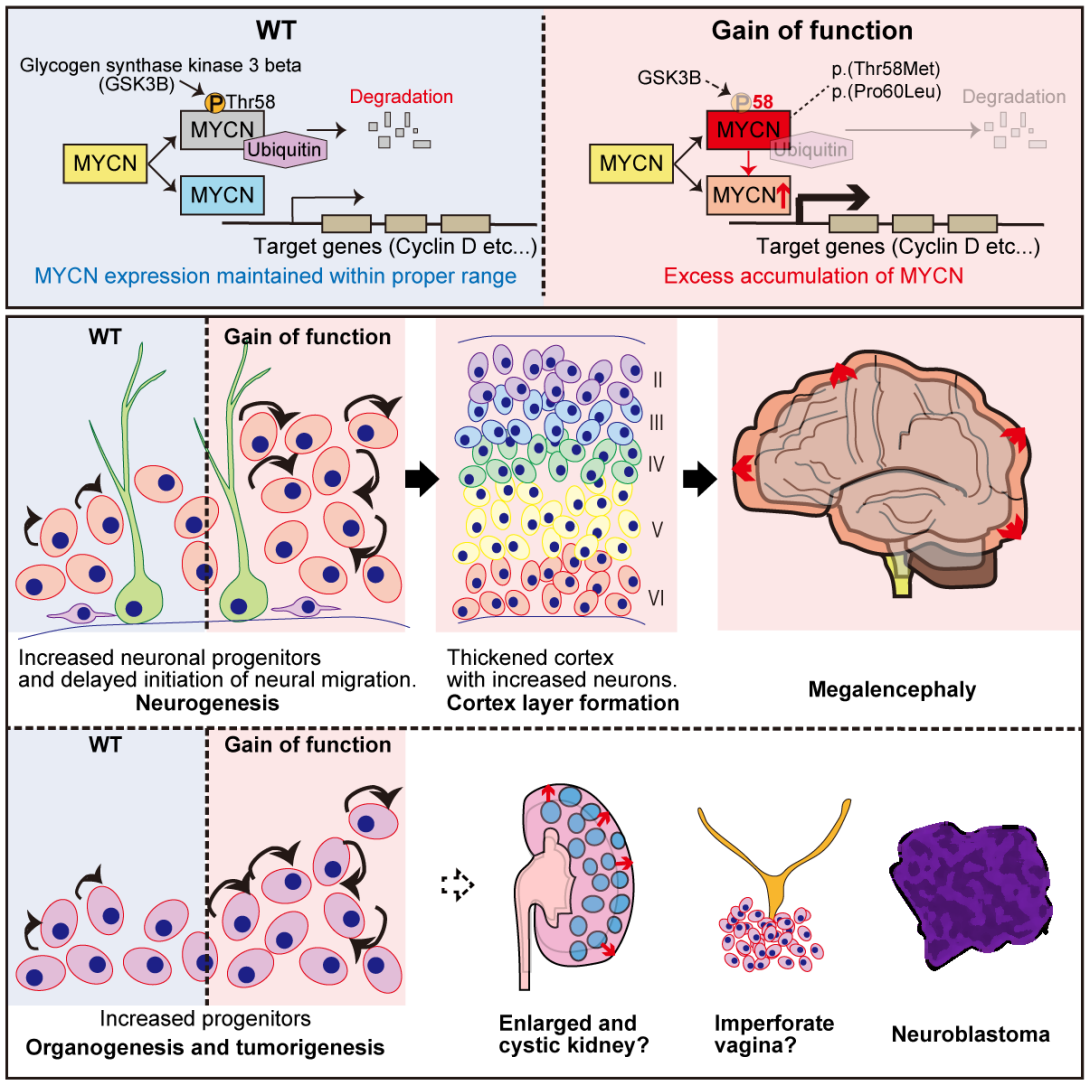
Graphical overview of MYCN gain-of-function induced megalencephaly-polydactyly syndrome. This figure presents findings on MYCN’s role in megalencephaly-polydactyly syndrome, highlighting gain-of-function *MYCN* variants linked to decreased protein degradation and increased cell proliferation. Through functional analyses and mouse models, the study reveals that these gain-of-function mutations lead to over-proliferation of neural precursors and morphological anomalies in kidneys and the female reproductive tract. The findings confirm MYCN’s significant proliferative role in both organogenesis and tumorigenesis, establishing a syndrome that contrasts with Feingold syndrome, which is associated with MYCN loss-of-function mutations.

We previously established the GoF MYCN-induced megalencephaly-polydactyly syndrome (34, 35) (summarized in **Figure 3**). For the functional aspect, HEK293T cells transfected with plasmids expressing MYCN-WT, MYCN-T58M, and MYCN-P60L revealed significantly lower T58 phosphorylation levels in those transfected with both MYCN-T58M and -P60L compared to MYCN-WT (34, 35). Additionally, when plasmids expressing MYCN-WT, MYCN-T58M, and MYCN-P60L were co-transfected with Fbw7, the ubiquitin ligase targeting MYCN, both MYCN-T58M and -P60L displayed increased stability and resistance to degradation (34, 35). Crucially, the mutant MYCN proteins retained their ability to activate transcription of downstream genes (*CCND1* and *CCND2*), affirming their sustained canonical activity as transcription factors, revealing GoF characteristics of both variants (34, 35).

Mouse models were generated to delve deeper into Mycn gain- and loss-of-function roles in murine development (35). The GoF (T58M/WT) mice recapitulated megalencephaly-polydactyly syndrome, while the haploinsufficient model (frameshift/WT) manifested microcephaly, which is one of the significant characteristics of Feingold syndrome (35). Semi-quantitative analysis unveiled decreased T58 phosphorylation and increased Mycn accumulation in GoF mice. Further investigations into the central nervous system at P0 and P7 elucidated a thickened cerebral cortex with increased neurons in GoF mice, contributing to megalencephaly, indicating that the megalencephalic phenotype in GoF mice was already determined prenatally (35).

The study then analyzed back neurogenesis in GoF mice, uncovering delayed neuronal migration and an elevated number of intermediate neural precursors at E14.5, contributing to the pathophysiological background of megalencephaly (35). The increased number of intermediate neural precursors (IPCs) and delayed neuronal migration were consistent with prior research indicating that Mycn overexpression leads to delayed cell cycle exit and migration. More precisely, MYCN has been reported to modulate the equilibrium between symmetrical and asymmetrical cell division, with overexpression promoting symmetrical cell division (98, 99).

Beyond the central nervous system manifestations, morphological alterations in various organs of GoF mice were observed, including digits, female reproductive system, and kidneys, which was consistent with its almost ubiquitous expression pattern (35). In the digits, over 90% of the mice exhibited postaxial polydactyly, which could be explained by the prior research that has elucidated the involvement of Shh signaling in limb development, influencing the establishment of the anterior-posterior axis for digit specification and the proliferation of limb mesenchymal cell (100–103).

Within the female reproductive system, imperforate vagina and hydrometra, arising from the vaginal closure with over-proliferative epithelium, were identified (35). Shh signaling is recognized for its role in activating the proliferation of Müllerian epithelial cell in the uterus and vagina (104), potentially contributing to the histological alterations observed in the female reproductive tract in our mouse model. Furthermore, the Shh signal is implicated in the progression of polycystic kidney disease (105), and its inhibition has demonstrated preventive effects on excessive proliferation and microcyst formation in the disease (106).

### MYCN-mediated transcribed genes model

The triple knockout mice of *Ccnd1*, *2*, and *3* were generated to investigate the role of cyclin Ds during development (107). The mice were viable until mid or late gestation but died due to a heart defect associated with severe hematologic abnormality, anemia, suggesting important roles in expanding hematopoietic stem cells. Fibroblasts deficient of Ccnds proliferated almost normally but required increased mitogenic stimulation in cell cycle reentry (107). The GoF *CCND2* variants, the cause of MPPH3, were clustered around a residue that is phosphorylated by glycogen synthase kinase 3β (GSK3β) (28). Mutant CCND2 prevents itself from proteasomal degradation compared to wildtype CCND2. *In utero* electroporation of mutant *CCND2* into embryonic mouse brains resulted in the transfected progenitors proliferating more and exhibited delayed cell cycle exit compared to cells transfected with wildtype *CCND2*, suggesting important functions in regulating cell cycle progression (28).

### Other MYCN-related genes model

The GoF *MAX* variants, the cause of polydactyly-macrocephaly syndrome, are present in the b-HLH-LZ domain, where the mutant MAX binds its target E-box sequence with a lower apparent affinity, leading to a more efficient heterodimerization with Myc and an increase in transcriptional activity of Myc (29).

Speaking of *FBXW7*, Fbw7 knockout mice exhibited hematopoietic and vascular development deficiencies, leading to early embryonic lethality (108, 109). The absence of Fbw7 resulted in elevated levels of cyclin E and increased notch signaling, emphasizing its regulatory functions in cell cycle control and notch pathway modulation (109). Consistently, cellular analysis with HEK293T cells transfected with the pathogenic variants of *FBXW7* showed an impaired ability to ubiquitinate and degrade substrates such as CCNE1 and CCNE2 (30).

## Discussion

Our comprehensive review sheds new light on the dual roles of MYCN in developmental processes as well as well-established roles in the context of oncogenesis. It should be emphasized that the intricate balance of MYCN expression is fundamental for normal development. The reduced expression leads to FS1 (92, 93) and excessive stability results in megalencephaly-polydactyly syndrome in developmental scenarios (34, 35). The importance of this intricately controlled balance is reinforced by the partially overlapped phenotypes of MYCN-related disorders and from the fact in the oncogenic field that dysfunctions of all the MYCN-related genes introduced in this review are associated with tumorigenesis and progression: hedgehog signaling (110–113), cyclin Ds (114–116), FBXW7 (117–119), and MAX (6).

The proper spatiotemporal expression of MYCN, both quantitatively and geographically, accomplished both by the precisely regulated transcription by the upstream pathways and intricately adjusted phosphorylation followed by ubiquitination by FBXW7 is key to normal developmental processes (120). Needless to mention the direct aberration affecting the quantity of MYCN expression, the dysregulation of its pathway component exerts adverse effects enough to compromise the normal developmental processes.

The strength of this review is that the MYCN’s direct contributions to the pathogenesis of human developmental diseases are made clear. This could result in the detailed understanding of the disorders associated with MYCN, such as SHH or Wnt/β-catenin pathway, contributing to the development of the therapeutic approaches targeting this specific pathway. Noteworthy, the growing evidence of the therapeutic approaches is proposed in terms of the treatment for MYCN-associated tumors, which targets MYCN transcription, stability, MYCN cofactors/coregulators and MYCN downstream targets (121). In addition, the investigations targeting hedgehog signaling, cyclin D, and FBXW7 are also under development (122–125). Though its relative difficulty for application to developmental disease due to the more intricate adjustment during development, not only quantitatively but also geographically, this oncologic knowledge might contribute to the development of therapeutic approaches even to the developmental disorders discussed in this review.

This review has potential limitations. Firstly, the disorder with GoF MYCN was just discovered that the clinical and molecular information is limited so far. For explaining the MYCN-related pathogenesis in associated disorders, it should be based not only on clinical information but also on molecular data. The future study would enrich the molecular understanding of the diseases, resulting in the comprehensive understanding of associated disorders. Secondly, we lack the data regarding MYCN’s transcriptional signature for the GoF model. It is unclear whether “enhancer invasion” would be present in the model in our situation where the expression level would be intermediate compared with MYCN amplification, The comprehensive understanding of the transcriptional signature is required to understand the pathophysiology more specifically.

## Conclusion

The dual roles of MYCN in development and oncogenesis underscore the significance in cellular proliferation, differentiation, and apoptosis. The intricate network of interactions and signaling pathways involving MYCN and its associated genes highlights the complexity of regulatory mechanisms governing normal development and their potential disruption in diseases. Continuing exploration of these pathways holds promise for unraveling the molecular backgrounds of developmental disorders and cancers, offering hope for new therapeutic strategies. Our review advances our understanding of MYCN’s role in human biology and opens new avenues for research into the prevention and treatment of the conditions associated with its dysregulation.

## Author contributions

YN: Writing – original draft, Writing – review & editing. KK: Writing – original draft, Writing – review & editing. HO: Writing – review & editing. YT: Writing – review & editing. SS: Writing – original draft, Writing – review & editing.
